# Circulating Cell-Free DNA Methylation Profiles in the Early Detection of Ovarian Cancer: A Scoping Review of the Literature

**DOI:** 10.3390/cancers13040838

**Published:** 2021-02-17

**Authors:** Xiaoyue M. Guo, Heather Miller, Koji Matsuo, Lynda D. Roman, Bodour Salhia

**Affiliations:** 1 Division of Gynecologic Oncology, Department of Obstetrics and Gynecology, Keck School Medicine of University of Southern California, Los Angeles, CA 90033, USA; xiaoyue.guo@med.usc.edu (X.M.G.); heather.miller@med.usc.edu (H.M.); 2 Division of Gynecologic Oncology, Department of Obstetrics and Gynecology, Norris Comprehensive Cancer Center, Keck School Medicine of University of Southern California, Los Angeles, CA 90033, USA; koji.matsuo@med.usc.edu (K.M.); lroman@med.usc.edu (L.D.R.); 3 Department of Translational Genomics, University of Southern California, Los Angeles, CA 90033, USA; 4 Norris Comprehensive Cancer Center, Keck School Medicine of University of Southern California, Los Angeles, CA 90033, USA

**Keywords:** ovarian cancer, early detection, biomarkers, cell-free DNA, liquid biopsy, epigenetics, methylation, scoping review

## Abstract

**Simple Summary:**

There are limited non-invasive methods for detecting epithelial ovarian cancer despite early detection and treatment dramatically increasing survival. As alterations in serum or plasma cell-free (cf)DNA methylation occur early in cancer development, they are promising biomarkers for ovarian cancer. Our literature review includes 18 studies depicting a wide array of gene targets and techniques. The data suggest a good performance of these cfDNA methylation tests, with accuracies up to 91% in detecting ovarian cancer in serum or plasma.

**Abstract:**

Epithelial ovarian cancer is the most lethal gynecologic malignancy and has few reliable non-invasive tests for early detection or diagnosis. Recent advances in genomic techniques have bolstered the utility of cell-free DNA (cfDNA) evaluation from peripheral blood as a viable cancer biomarker. For multiple reasons, comparing alterations in DNA methylation is particularly advantageous over other molecular assays. We performed a literature review for studies exploring cfDNA methylation in serum and plasma for the early diagnosis of ovarian cancer. The data suggest that serum/plasma cfDNA methylation tests have strong diagnostic accuracies for ovarian cancer (median 85%, range 40–91%). Moreover, there is improved diagnostic performance if multiple genes are used and if the assays are designed to compare detection of ovarian cancer with benign pelvic masses. We further highlight the vast array of possible gene targets and techniques, and a need to include more earlier-stage ovarian cancer samples in test development. Overall, we show the promise of cfDNA methylation analysis in the development of a viable diagnostic biomarker for ovarian cancer.

## 1. Introduction

Epithelial ovarian cancer (OC) is the most lethal gynecologic malignancy with a 5-year survival rate under 50%. Although disease confined to the ovaries is associated with a greater than 90% survival rate, the majority of patients who present with distant metastasis have a dismal prognosis [1,2]. There is also an increasing age-standardized incidence rate of ovarian cancer worldwide [3], while mortality rates have remained relatively constant [2].

Most epithelial OCs have high-grade serous histology, an aggressive subtype with few known risk factors, the majority of which present with advanced-stage disease and have a poor prognosis. Cancer-antigen 125 (CA125), which is elevated in up to 90% of advanced-stage disease [4], remains the most commonly used marker for monitoring high-grade serous OC [5,6]. However, CA125 has several shortcomings including poor sensitivity for early-stage cancers and poor specificity given its elevation in many other conditions [7]. The Food and Drug Administration (FDA) has approved various new serum biomarkers and biomarker panels (i.e., human epididymis protein 4, Aspira Labs’ OVA1 and Overa comprising CA125, apolipoprotein A1, beta-2 microglobulin, transferrin, and pre-albumin) as well as multimodal tests including transvaginal ultrasounds (i.e., the risk of malignancy index or Roche Diagnostics’ risk of ovarian malignancy algorithm) [8]. Unfortunately, these approaches have been largely inadequate as they have not yielded a shift in the diagnosis of OC, especially at the earlier stages. In addition, they lack the sensitivity and specificity to be considered screening tests [7,9] and are thus not currently recommended for clinical use by any governing organization.

Recent developments in proteomics and genomics have produced novel non-invasive methods for cancer detection and screening. Technological advances have led to the discovery of many promising tumor-associated autoantibodies and serum/plasma protein markers, but unfortunately most markers are only useful for late-stage disease [10]. Genomic techniques might be more valuable as early tumor-related gene alterations can be detected with cell-free (cf) DNA and RNA. Tumor DNA was initially found to be circulating in the serum/plasma of cancer patients over 40 years ago; hypotheses for this phenomenon remain limited and range from lysis of cells at the tumor–circulation interface to apoptosis of tumor cells [11]. Researchers are searching for either alterations in DNA or microRNA that suggest early expression changes towards malignancy, or for actual fragments of circulating tumor DNA [9,11]. The “liquid biopsy” potential of cfDNA has gained increasing interest and attention for screening, diagnosis, treatment, and monitoring in the spectrum of human malignancies [12]. The past decade has seen a surge in the number of studies evaluating the diagnostic potential of cfDNA, with meta-analyses showing sensitivity and specificity of 0.70 to 0.90 in detecting OC from non-OC samples, respectively, with area under the curve (AUC) values of 0.90 [13,14]. Notably, more recent studies show steady improvements in diagnostic accuracy, and subgroup analyses of systematic reviews suggest that epigenetic markers are particularly more effective over other quantitative detection methods of cfDNA concentrations or degrees of chromosomal instability [14]. 

Of all mechanisms for epigenetic DNA modification, methylation alterations are the most common. DNA methylation occurs when a methyl group is added to a cytosine base in a cytosine–phosphate–guanine (CpG) dinucleotide, thus controlling gene transcription and expression. There are several advantages to utilizing aberrant DNA methylation over other molecular alterations such as point mutations or serum/plasma-based protein markers. For one, DNA methylation changes occur early in tumorigenesis and are highly chemically stable markers [15,16,17]. The frequency and distribution of aberrantly methylated DNA further enhances its detection sensitivity [16,18,19]. Moreover, DNA methylation measurements incorporate numerous regions, each with multiple CpG positions, allowing better limits of detection than for protein-based markers or DNA mutations. These aberrant CpG alterations also rarely occur in normal cells, so the signal can be detected with a notable degree of sensitivity, even in the presence of background methylation derived from normal cells [19,20,21,22,23]. Finally, large-scale DNA methylation alterations are tissue- and cancer type-specific and therefore potentially have greater ability to detect and classify cancers in patients with early-stage disease [24,25]. Methods for detecting DNA methylation are varied and several technologies currently exist. The most widely used method involves bisulfite conversion of unmethylated cytosine to uracil, which allows subsequent identification of the non-converted CpGs by imposing a cytosine–thymine substitution of unmethylated cytosines [26]. 

However, despite increasing investigation on DNA methylation biomarkers for OC, their use is still novel, and a comprehensive review does not exist. We thus conducted a scoping review of the literature for cfDNA methylation alterations in serum/plasma for early detection of ovarian cancer. Specifically, we compiled methodological and outcome data from the included studies to compare and summarize the state of research in this emerging research landscape.

## 2. Results

**Figure 1:**
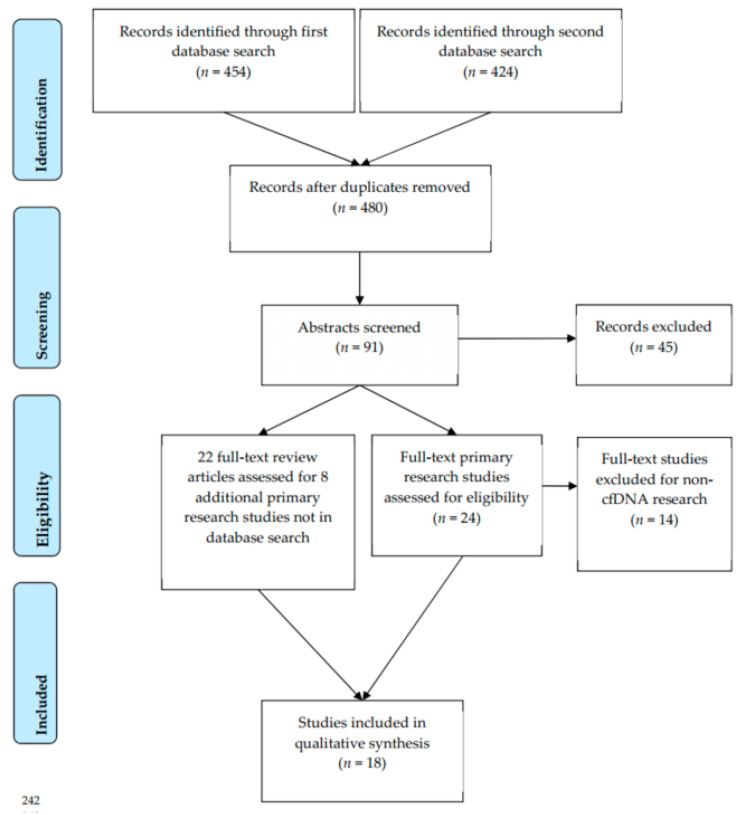
PRISMA flow diagram.

After removal of duplicates, 480 article titles were screened for thematic relevance over the two database searches. Subsequently, 91 articles underwent abstract review. We selected 24 primary research studies and 22 review articles for full-text review. After full-text review, 18 primary research studies were included for review, including 8 studies abstracted from previously published review articles. Full-text primary research articles were excluded from final analysis for evaluating methylation in sources that were not serum- or plasma-based cfDNA, including leukocyte-derived DNA from peripheral blood (*n* = 6), primary ovarian tissue (*n* = 5), the cervix (*n* = 2), and the endometrium (*n* = 1). A flowchart is shown in Figure 1.

### 2.1. Study Characteristics 

The 18 articles were published between 2004 and 2018 (Table 1). Overlapping investigators were noted in three pairs of studies [27,28,29,30,31,32], with one pair using samples from the same patient and control populations [29,30] and another pair using the same control population [31,32]. The case–control study design was utilized by all studies except for one case series [33] and two that analyzed the diagnostic potential of their chosen methylation targets with prospective samples [34,35]. Most studies used serum samples for their cfDNA extraction, while six used plasma [27,28,30,31,32,36] and one did not specify serum or plasma [37]. Cohort sizes used for serum/plasma diagnostics ranged from 21 to 164 (median 46, interquartile range (IQR) 33.8–59.8) from ovarian cancer patients and 8 to 150 (median 30, IQR 20–51) from healthy controls; 10 studies included a separate comparison arm of serum/plasma from patients with benign ovarian or pelvic masses (i.e., endometriomas, ovarian cysts, cystadenomas, fibroids; median 30, IQR 14–119). 

All studies except one [35] included serum/plasma only from patients with epithelial OC; the most common histology was serous (median 60% of the samples per study, IQR 50–100%), followed by endometrioid (median 11%, IQR 0–17.5%) and mucinous (median 10%, IQR 0–14.5%). Serum/plasma samples were also predominantly from advanced-stage (III–IV) ovarian cancer patients (median 77.5% of samples per study, IQR 51.5–87.8%). No histology or staging data were provided for two publications [38,39].

### 2.2. Methylation Modification and Analysis

Most studies utilized bisulfite conversion as the basis for DNA methylation analysis with a variety of proprietary kits: Four studies used Qiagen’s EpiTect kit [30,34,42,45], three used Zymo’s EZ DNA kit [31,32,40], two used CpGenome’s kit [41,43], and one each used Epigentek’s MethylAmp [38] and BisulFlash [44] kits. Three groups performed bisulfite conversion using traditional laboratory techniques with NaHSO_3_ [33,36,37], one group had an outside institution conduct the bisulfite conversion [35], and two used a self-developed technology for genome-wide DNA methylation analysis called “MethDet” [27,28]. One study did not specify how methylation evaluation was conducted [29].

Subsequent methylation analysis was mostly conducted using methylation-specific polymerase chain reaction (MSP) followed by gel electrophoresis for comparison, with some studies reporting slight PCR modifications. Other techniques employed include two studies using the self-developed “MethDet” technique to analyze their products via custom-designed methylation assay microarrays [27,28], two studies utilizing sequencing-specific information for methylation quantification, and one study using a methylation-sensitive high-resolution melting analysis assay for quantitative estimation of methylation [31].

### 2.3. Target Genes

All studies except three had preselected genetic targets for methylation detection based on prior studies or literature reviews. The remaining three studies performed discovery analysis to determine the most suitable candidate genes: Two studies used their own microarray of 56 promotor fragments to determine which genes were differentially methylated between clinical cohorts [27,28], and one study performed genome-wide methylation analysis with subsequent validation of a small subset of the best candidate markers [35]. 

A total of 26 genes or gene families were evaluated by the various publications comparing OC with non-OC specimens (Table 2). Eleven studies evaluated the methylation profiles of single genes, while seven studies evaluated a panel of genes (median 4, range 3–7). Most gene targets were tumor suppressors, and 10 were utilized by more than one study. The gene included most often was *RASSF1A,* a tumor suppressor involved in numerous apoptosis and cell cycle checkpoint functions. *OPCML,* encoding for a plasma membrane protein involved in cell-to-cell recognition and adhesion, was the next commonly targeted gene. Most studies evaluated methylation in the promotor region of the gene; two studies [34,38] alluded to, but did not explicitly state, use of the promotor region(s), while two studies [30,46] did not specify where methylation occurred in their chosen gene(s).

### 2.4. Diagnostic Performance

As summarized in Table 1 and Supplemental Table S1, studies had wide ranges for the performance(s) of the targeted genes and utilized an array of controls and comparisons. All but one study reported relevant statistical results or provided enough data to extrapolate some statistical measures. Studies evaluated serum/plasma methylation alterations in differentiating OC patients from healthy controls (39%, 7/18 studies), benign ovarian masses (11%, 2/18 studies), or both (39%, 7/18 studies). Two studies only compared serum/plasma with paired tissue methylation [33,42].

**Figure 2:**
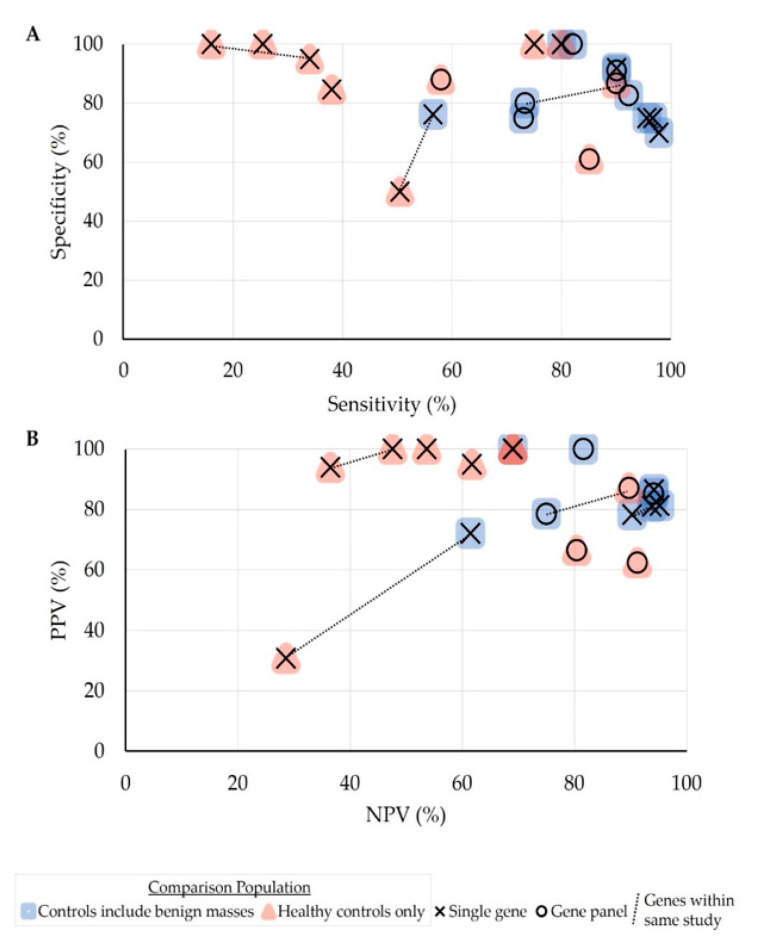
Diagnostic performance of targeted genes in differentiating ovarian cancer from controls by (**A**) sensitivity and specificity or (**B**) positive predictive value (PPV) and negative predictive value (NPV). Data points depict separate gene comparisons. Colored shapes represent the control population used in each comparison: Healthy patients plus those with benign masses (blue squares) or healthy patients only (red triangle). Symbols represent whether a single gene (X) or multiple genes (O) were included in the methylation panel. Lines connect genes that were evaluated in the same study.

For all studies, sensitivities ranged from 16 to 97.8% (median 80%), specificities ranged from 50 to 100% (median 86.7%), and accuracies ranged from 40 to 91.2% (median 84.8%). In general, the positive predictive value (PPV) of each study’s reported methylation tests in determining OC was higher than the negative predictive value (NPV). The average accuracies tended to be higher when the methylation profiles of a panel of genes were evaluated rather than a single gene (89.9% vs. 74.5%, *p* = 0.07) and when using both benign ovarian masses and healthy controls as the comparison arm against OC instead of just using healthy controls (85.4% vs. 64.8% *p* = 0.006; Figure 2). Six studies found improved discriminatory abilities with serum/plasma from advanced-stage OC patients compared to those with early stages [27,29,30,43,44,45], but most reported no correlation with clinicopathologic characteristics. Whether serum or plasma was used for cfDNA extraction and the subsequent bisulfite conversion kit or methylation analysis technique employed did not lead to significant differences in test characteristics. Wang et al. reported the best overall diagnostic performance in their two studies, which used the same patient population but had slightly different gene targets for methylation analysis: The earlier study [29] looked at a panel of genes (*RUNX3*, *TFPI2*, and *OPCML* (accuracy 90.7%) in differentiating OC patients from healthy controls or patients with benign ovarian masses, while their later study [30] narrowed down their analysis to *OPCML* (accuracy 91.2%). When comparing individual genes (Supplemental Table S1), *OPCML* also had the greatest overall sensitivity and specificity. 

## 3. Discussion

To our knowledge, this study represents the first scoping review of serum/plasma cfDNA methylation targets for the early diagnosis of OC. We included 18 studies depicting how epigenetic alterations could be used to diagnose OC patients of differing stages and histologies with diagnostic accuracies up to 91.2% (median 84.8%, range 40–91%), in line with prior meta-analyses of using cfDNA in general for OC [13,14]. Better diagnostic performance was appreciated for studies that included benign masses as comparison arms instead of only healthy controls and when multiple genes were evaluated for methylation status instead of a single gene. 

The inclusion of benign masses is a clinically relevant comparison given the clinical dilemma of undiagnosed pelvic masses. The improved accuracy noted when these benign masses were used as a comparison group is perhaps a statistical result of studies including benign masses having larger overall cohort sizes or due to a real epigenetic difference between the cfDNA methylation patterns of benign and malignant ovarian masses. Unfortunately, the nuances behind this differentiation could not be elucidated in our current review and warrant further evaluation. Studies also reported a wide range of genetic targets, including classic tumor suppressor genes such as *BRCA1* and *PTEN* and OC-specific tumor suppressors such as *RASSF1A* and *OPCML*. Since most genes were only included in one or two studies, our ability to conduct gene-level analysis from this review was very limited. Moreover, as no single gene has been identified as being predominantly methylated for ovarian cancer tissues [47], including a panel of methylated gene targets would appropriately be more suitable. This is concordant with both our results and studies in ovarian and other cancers showing that utilizing multimodal methods and algorithms provides improved screening and diagnostic performances [7,9,48]. Indeed, non-invasive methylation-based biomarker tests that are currently commercially available for various cancers tend to be panels that target multiple genes [17,49,50]. 

A few studies in our review directly compared the performance of cfDNA methylation status with CA125 and found better detection using methylation methods [29,30,34,45]. For example, Widschwendter et al. [35] stratified their screening population by CA125 levels and found improved accuracy of their methylation test when only analyzing healthy controls with normal CA125. Prospective trials will ultimately be needed to determine the comparative effectiveness of these tests; future studies would also benefit from utilizing a combination of traditional biomarker, ultrasonography, and epigenetic profiles to further enhance early diagnosis potential. 

Given the known challenges in obtaining sufficient cfDNA for adequate analysis from healthy individuals and those with early-stage cancers [12,51], it was promising to see that all but one study included patient samples from early-stage OC. However, samples were still predominantly from stage III and IV patients. This expectedly led to better discriminatory abilities of the methylation tests with advanced-stage patients, although two studies [30,34] found improved detection of early-stage OC using methylation markers as compared to CA125. Although small yields of cfDNA extractable from peripheral blood will continue to be a barrier to the validation of methylation methods, technological advancements will assist in overcoming this technical hurdle; in clinical practice, larger volume blood draws can also offset the low cfDNA yields. Until then, other techniques currently being explored include methylation analysis of bodily fluids closer to the ovaries such as endometrial lavages, endocervical brushings, Papanicolaou smears, and vaginal fluids [4,9]. 

Overall, the wide range of reported sensitivities and specificities for the varying studies is possibly a function of their assorted techniques, gene targets, and human samples used. Five separate commercially available bisulfite conversion kits were used likely due to their availability for each research group, which may have uncertain downstream effects on analysis given the differences in recovery or conversion rates between the kits [52]. Furthermore, while serum samples tend to produce greater cfDNA yields than plasma, they have poorer fidelity in maintaining mutation frequencies because of dilution with non-cancer cfDNA [53,54]. However, we did not note better diagnostic performances for certain methods or detect a difference between those studies using serum or plasma. 

As such, the main limitation of this review lies in the heterogeneity of the included studies, which precludes robust conclusions from being drawn. This is a function of the relative novelty of epigenetic evaluation of cfDNA, as evident by most of the included studies being published in the past decade and the lack of standardized methylation analysis methods. Many of the included studies were exploratory and retrospective with small sample sizes, but the two with prospective screening samples showed promising results with accuracies above 80% [34,35]. We may have also missed relevant studies from excluding non-English publications, such as one Chinese language study that was included in a previous systematic review on cfDNA [55]. However, our current review ultimately provides the most current evaluation of the state of methylation science for OC and demonstrates exciting future possibilities of serum/plasma cfDNA methylation in assisting with diagnosis and early detection.

## 4. Materials and Methods 

### 4.1. Search Strateg 

A systematic search was performed following the criteria of Preferred Reporting Items for Systematic Reviews and Meta Analyses-Extension for Scoping Reviews (PRISMA-ScR) [56] in PubMed’s Medline and Elsevier’s Embase and Scopus. Articles were required to be in English, with no limitation on the date of publication. MeSH terms were used for PubMed but were unavailable for use in Elsevier searches. The initial query was made on July 8 2020 and a second broader search was performed on September 10 2020 utilizing the same databases with a slight variation in search terms that removed the requirement for biomarkers (Table 3).

### 4.2. Inclusion and Exclusion Criteria

Articles were required to be primary, original research evaluating the DNA methylation profiles of cell-free DNA in peripheral blood for patients with OC. Due to the relatively novel nature of the research, we placed limited restrictions on the study size, the presence of controls, techniques utilized, or the diagnostic performance of the described epigenetic marker. Excluded publication types included conference abstracts, letters, or editorials without complete methods or data. Review articles identified through the systematic searches were excluded if they did not contain original research but examined for additional studies.

### 4.3. Data Extraction

Eligible articles were examined for basic study characteristics including publication year, sample size(s) and source for cases and controls, clinicopathologic information for the OC cases (histological subtype and tumor stage, if available), method for DNA methylation analysis, and gene target(s). Key findings including diagnostic performance markers for the genetic biomarkers were also retrieved; this included any available sensitivities, specificities, positive and negative predictive values (PPV and NPV), accuracies or areas under the ROC (receiver operating characteristic) curve, and associated *p*-values. Finally, a summary section highlighted the given study’s serum or plasma DNA methylation analysis in differentiating or detecting OC along with any comparison groups. If the study did not provide explicit summary statistics, extrapolated summaries were calculated based on available data based on the following calculations: Sensitivity [True Positive (TP)/(TP + False Negative (TN)], specificity [True Negative (TN)/(False Positive (FP) +TN)], accuracy [(TP+TN)/(TP + FP + TN + FN) ], PPV [TP/(TP+FP)] and NPV [TN/(TN + FN)]. A data extraction sheet was developed with the above variables and actively refined during a first-pass review of the articles. All articles were then reviewed a second time to ensure that the included information was consistent. Any ambiguities in the retrieved data were resolved by consensus amongst the co-authors.

Diagnostic data were compiled and analyzed with Microsoft Excel, Version 1908. Mean ± standard deviation, median, and range were calculated as continuous variables. Comparisons were made with independent sample t-tests for continuous variables. All analyses assumed a two-sided 5% level of significance.

## 5. Conclusions

In recent years, multi-cancer screening blood tests utilizing cfDNA are gaining attention and funding but suffer from detection rates around 50% and lack of large prospective clinical data [25,57]. Commercial tests for specific cancers such as colorectal and liver are also coming to fruition, with many using DNA methylation techniques [50]. For ovarian cancer, a minimally invasive diagnostic marker would be invaluable. However, despite ample attempts at studying biomarkers and algorithms, efforts have fallen short in identifying a reliable strategy for the detection of ovarian cancer [4]. As such, the nature and time course of DNA epigenetic alterations make methylation status a unique and robust biomarker compared to other liquid biopsies specifically for early cancer detection. Our review shows the current breadth of research in this topic and suggests improved diagnostic performance of methylation tests when comparing OC with benign pelvic masses and when including multiple genes in the analysis. We also highlight the vast array of possible gene targets and techniques and a need to include more earlier-stage OC samples in test development. Overall, we show the promise of cfDNA methylation analysis as a viable diagnostic biomarker for OC.

## Figures and Tables

**Table 1:** Summary of included literature review studies.

First Author, Year	Source	Cases			# Controls	Methylation Method	Gene Target(s)	Findings and Study Summary
		Histology (%)	Stage (%)	# Cases				
De Caceres 2004 [37]	Tissue, serum or plasma, peritoneal fluid	Tissue and serum/plasma: Serous (65), endometroid (12), mucinous (8), clear cell (10), transitional cell (2), undifferentiated (2) Tissue only samples: Unknown	Tissue and serum/ plasma: I (20), III (62), IV (18) Tissue only samples: I (100)	Tissue and serum/ plasma: 40 OC 10 borderline Tissue only archival samples: 21 OC	Tissue and fluid: 10 benign ov mass 10 normal ov/peritoneal fluid Serum/ plasma: 20 healthy controls	Bisulfite modified with NaHSO_3_; MSP	*RASSF1A, BRCA1, APC, p14-ARF, p16-INK4a, DAPK*	Tissue: -99% OC and borderline (70/71) had hypermethylation of at least one of the 6 genes, in all stages and histologic types Comparisons: -82% (41/50—OC and borderline) of matched serum/plasma and tumor had identical hypermethylation status, including 76.5% (13/17) stage I and 84.8% (28/33) stage III-IV → Sensitivity 82% -0% non-hypermethylated tumors had hypermethylated serum -0% (0/40) hypermethylation in tissue, peritoneal fluid, or serum for benign ov msas, normal ov, or controls → Specificity 100% -No correlations between methylation and stage or histology ----------------------- Summary for plasma/serum test in differentiating OC from benign/control: -Sensitivity 82% (41/50 for OC and borderline), specificity 100% (40/40 healthy controls, benign ov mass, normal ov) -*PPV 100% (41/41), *NPV 81.6% (40/49), *accuracy 90% (81/90)
Melnikov 2009 [28]	Tissue, plasma	Tissue: Serous (70), endometroid (30) Plasma: Serous (100)	Tissue: I-II (13); III-IV (87) Plasma: III–IV (100)	Tissue: 30 OC Plasma: 33 OC	Tissue: 30 healthy ov (RRSO) Plasma: 33 healthy controls	Methylation analysis with MethDet technique	Selected after MethDet analysis For tissue: *BRCA1, EP300, NR3C1, MLH1, DNAJC15, CDKN1C, TP73*, *PGR, THBS1, PYCARD* For plasma: *BRCA1, H1C1, PAX5, PGR, THBS1*	Tissue: -Sensitivity, specificity, and accuracy of methylation test (averaged after 25 rounds of cross-validation) in detecting OC vs. heathy ov tissue: 69.4% (20.82/30), 70.2% (21.06/30), 69.8% (41.88/60) Plasma: -Sensitivity, specificity, and accuracy of methylation test (averaged after 25 rounds of cross-validation) in detecting OC vs. healthy control serum: 85.1% (28.25/33), 61.1% (20.16/33), 73.1% (48.24/66) ----------------------- Summary for MethDet plasma test in differentiating OC from controls: -Sensitivity 85.1% (28.08/33), specificity 61.1% (20.16/33), -PPV 68.6% (28.02/40.85), NPV 80.4% (20.16/25.08), accuracy 73.1% (48.24/66)
Su 2009 [40]	Tissue, serum	Tissue: Serous (66), mucinous (26), endometrial (6), other (2)	Tissue: I (25); II (9); III (56); IV (10) Serum subset: No breakdown	Tissue: 126 OC 14 borderline Serum subset: 26 OC	Tissue: 75 benign ov mass or normal ov Serum subset: 20 benign ov mass	Bisulfite modified with EZ DNA kit; MSP	*SFRP1, SFRP2, SFRP4, SFRP5, SOX1, PAX1, LMX1A* Plasma: Excluded *SFRP4*	Tissue: -Methylation rates -OC: *SFRPI* (35%), *SFRP2* (63%), *SFRP4* (2%), *SFRP5* (44%)*, SOX1* (59%)*, PAX1* (50%)*, LMX1A* (35%) -Lower rates for borderline compared to OC; lowest rates for benign ov mass (*p* < 0.001 for all comparisons except *SFRP4*) -No correlations between methylation with stage or grade Plasma (no numbers provided for calculating statistics): -Significant concordance between plasma and tissue methylation for all markers—highest concordance with *SFRP1* and *SFRP5* (*p* < 0.001) -Best potential for screening gene combinations with *SOX1, PAX1, SFRP1* ----------------------- Summary for serum test in differentiating OC from benign tumor for: -*SOX1 + PAX1 + SFRP1*: Sensitivity 73.1%, specificity 75% -All 6 genes: Sensitivity 73%, specificity 55%
BonDurant 2011 [33]	Tissue, serum	Serous (100)	Full/subset: I/II (4/5), III (83/71), IV (11/14), unk (2,10)	Tissue: 106 OC Serum subset: 21 OC	n/a	Bisulfite modified via NaHSO_3_; multiplexed real-time MSP assay	*RASSF1A*	Tissue: -51% (45/106) OC methylated; 25% (1/4) stage I-II Serum: -86% (18/21) OC methylated; 0% (0/1) stage I-II Comparison: -100% (18/18) of methylated serum samples had positive tumor methylation - Methylation correlated with increased age. No correlations between methylation with stage, clinical response, treatment, survival. ----------------------- Extrapolated summary for methylation assay in identifying OC for serum samples compared to tissue samples: -Sensitivity 86% (18/21), 100% concordance of serum with tissue testing for OC methylation status
Häfner 2011 [38]	Tissue, serum	Tissue: Serous (81), endometroid (12), papillary (16), clear cell (3), neuroendocrine (3) Serum subset: No breakdown	Tissue: IIc (6), III (78), IV (16) Serum subset: No breakdown	Tissue: 32 OC Serum subset: 23 OC	Tissue: 30 fibroid 20 healthy controls Serum subsets: 21 fibroid 8 healthy controls	Bisulfite modified via MethylAmp kit; MSP with sequencing	*DAPK*	Tissue: -Aberrant methylation: 50% (14/28) OC, 35.3% (6/17) fibroid Serum: -Aberrant methylation: 56% (13/23) OC, 23.8% (5/21) fibroid, 50% (5/8) controls -No correlations between serum methylation and clinicopathologic characteristics ----------------------- Extrapolated summary for serum test in differentiating OC: -From control: Sensitivity 56.5% (13/23), *specificity 50% (4/8), *PPV 30.9% (4/17), *NPV 28.6% (4/14), *accuracy 54.8% (17/31) -From fibroids: Sensitivity 56.5% (13/23), *specificity 76.2% (16/21), *PPV 72.2% (13/18), *NPV 61.5% (16/26), *accuracy 65.9% (29/44)
Liggett 2011 [27]	Plasma	Serous (100)	III (60), IV (40)	30 OC	30 benign ov mass 30 healthy controls	Methylation analysis with MethDet technique	Selected genes after analysis: OC vs. Control: *CALCA, EP300,* and *RASSF1A * Benign vs. control: *BRCA1, CALCA, CDKN1C* OC vs. benign: *PGR-PROX, RASSF1A*	Diagnostic performance of methylation test (uncertain how many runs were performed) in: -Differentiating OC from controls: Sensitivity 90.0%, specificity 86.7% -Differentiating benign ov mass from controls: Sensitivity 90.0%, specificity 76.7% -Differentiating OC from benign ov mass: Sensitivity 73.3%, specificity 80.0% ----------------------- Summary for MethDet plasma test in differentiating OC: -From controls: Sensitivity 90.0%, specificity 86.7%, PPV 87.1%, NPV 89.7% -From benign ovarian masses: Sensitivity 73.3%, specificity 80.0%, PPV 78.6%, NPV 75.0%
Dong 2012 [41]	Tissue, serum	Serous (50), endometrioid (17), mucinous (33)	I (8), II (3), III (52), IV (36)	36 OC	25 healthy controls	Bisulfite modified via CpGenome kit; MSP	*SLIT2*	Tissue: -Aberrant methylation: 80.6% (29/36) OC cases Serum: -Aberrant methylation: 93.1% (27/29) case-matched aberrantly methylated OC tissue; 0% (0/25) controls (p<.0001) -0% (0/7) of remaining non-aberrantly methylated tissue samples had aberrant serum methylation -No correlation between serum methylation with stage, histology, age, CA125 ----------------------- Extrapolated summary for serum test in differentiating OC from control: -Sensitivity 75% (27/36), specificity 100% (25/25) -PPV 100% (27/27), NPV 69% (25/36), accuracy: 85% (52/61)
Wang 2013 [42]	Tissue, serum	Serous (60), endometroid (20), clear cell (10), mucinous (10)	I (30), II (23), III (47)	60 OC	30 benign ov mass 30 normal ovary/ healthy controls	Bisulfite modified via EpiTect kit; real-time PCR	*BRCA1*	Tissue: -Hypermethylation almost 100% of stage II and III OC, higher frequency in stage III -Stage I not differentially methylated from stage II (*p* > 0.05) -Stage II more methylated than stage III, normal, and benign ov (*p* < 0.05) -Stage III more methylated than all groups (*p* < 0.01) Serum: -Hypomethylation in 100% of stage I, benign, and controls -Stage I not differentially methylated from stage II (*p* > 0.05) -Hypermethylation frequency higher in stage III OC than all groups (*p* < 0.05) -No correlations for methylation between controls with benign and stage I, stage I and stage II, or with histology ----------------------- Summary for serum test in identifying OC methylation compared to tissue test (no data for comparative statistics): -Serum less sensitive than tissue for methylation analysis -Higher methylation status in higher tumor stages
Zhang 2013 [34]	Serum	Serous (71), mucinous (12), clear cell (12), endometrioid (14), mixed (7), other (5) Screening: Serous (77), mucinous (10), endometrioid (8), other (5)	I (56) II (10) III (52) IV (1) Screening: I (15), II (10), III (74)	73 OC Screening: 69 pelvic masses (39 OC, 29 benign)	53 benign ov mass 62 healthy controls	Bisulfite modified via EpiTect kit; multiplex-PCR	*APC, RASSF1A, CDH1, RUNX3, TFPI2, SFRP5, OPCML*	-Diagnostic performance of methylation in differentiating OC from benign (unclear which numbers used for calculation): sensitivity 90.57%, specificity 89.66%, AUC 0.9126 -AUC early stage vs. benign: 0.8916; advanced stage vs. benign: 0.9313 -Prospective diagnosis with screening cohort in differentiating OC from benign (unclear which numbers for calculation): sensitivity 92.3%, specificity 89.9%, AUC 89.9% -AUC early stage vs. benign: 0.8218, advanced stage vs. benign: 0.9127 -No correlations between methylation status with stage or histology -Methylation status had stronger diagnostic performance than CA125 for early-stage OC patients (*p* = 0.004) but not advanced-stage OC patients (*p* = 0.6) ----------------------- Summary for serum test in differentiating OC from benign ov mass in prospective screening sample: -For all OC samples: Sensitivity 92.3%, specificity 82.7%, AUC 89.9% -For early-stage OC samples: Sensitivity 83.3%, AUC 82.2%, no specificity data -For advanced-stage OC samples: Sensitivity 93.9%, AUC 91.3%, no specificity data
Wu 2014 [36]	Tissue, plasma	Serous (49), mucinous (32), endometrioid (19)	Early (47), advanced (53)	47 OC	14 benign ov mass 10 normal ov	Bisulfite modified with NaHSO_3_ and hydroquinone; MSP	*RASSF1A*	Tissue: -51% (24/47) OC methylated, 0% (0/20) benign and normal Plasma: -36% (17/47) OC methylated, 0% (0/20) benign and normal -“Positive correlation” between serum and tissue methylation profiles -No correlations between methylation with clinicopathologic characteristics ----------------------- Extrapolated summary for plasma test in differentiating OC from benign ov mass or control: -Sensitivity 36.2% (17/47), specificity 100% (20/20) -PPV 100% (17/17), NPV 40% (20/50), accuracy 55.2% (37/67)
Zhou 2014 [43]	Tissue, serum	Serous (44), endometroid (22), mucinous (13), clear cell (11), undifferentiated (9)	I (16), II (7), III (62), IV (16)	Tissue and serum: 45 OC	Tissue: 40 normal ov Serum: 20 healthy controls	Bisulfite modified via CpGenome DNA Modification kit; MSP	*OPCML*	Tissue: -Hypermethylation in 87% (39/45) OC and 0% (0/40) normal ov Serum: -Hypermethylation in 80% (36/45) OC and 0% (0/20) healthy control -Correlation between methylation with increasing stage (*p* < 0.05). No correlations with histology. ----------------------- Extrapolated summary for serum test in differentiating OC from control: -Sensitivity 80% (36/45), specificity 100% (20/20) -PPV 100% (36/36), NPV 69% (20/29), accuracy 86.1% (56/65)
Zuberi 2014 [44]	Serum	Serous (46), mucinous (46), endometrioid (4), clear cell (2), undifferentiated (2)	Early (20), advanced (80)	50 OC	20 healthy controls	Bisulfite modified via BisfulFlash DNA Modification kit; MSP	*RASSF1A, PTEN*	-*RASSF1A* methylated in 34% (17/50) OC and 5% (1/20) healthy control 1/20 -*PTEN* methylated in 16% (8/50) OC and 0% (0/20) healthy control -Correlations between methylation with: menopausal status (*p* = 0.03) and histology (*p* = 0.03; highest correlation with serous) for *RASSF1;* higher stages for both gene targets (NS) ----------------------- Extrapolated summary for serum test in differentiating OC from control for: *-RASSF1A:* Sensitivity 34% (17/50), specificity 95% (19/20), PPV 94% (17/18), NPV 36.5% (19/52), accuracy 51.4% (36/70) *-PTEN:* Sensitivity 16% (8/50), specificity 100% (20/20), PPV 100% (8/8), NPV 47.6% (20/42), accuracy 40% (28/70)
Wang 2015 [29]	Tissue, serum	Serous (58), endometrioid (11), mucinous (10), clear cell (10), other (11)	I (46), II (8), III (44), IV (1)	71 OC	43 benign ov mass 80 healthy controls	Methylation modification via unknown technique; multiplex-nested MSP	*RUNX3, TFPI2, OPCML*	Diagnostic performance of methylation status in detecting all OC, early OC, and advanced OC: -Sensitivity: 90.14% (64/71), 84.62% (33/39), 93.75% (30/32) -Specificity: 91.06% (112/123) -PPV: 85.33% (64/75), 75%, 73.17% (30/41) -Methylation status had stronger PPV than CA125 -No correlation between methylation and stage ----------------------- Summary for serum test in differentiating: -All OC from controls/benign ov mass: Sensitivity 90.1% (64/71), specificity 91.1% (112/123), PPV 85.3% (64/75), *NPV 94.1% (112/119), accuracy 90.7% (176/194) -Early OC from controls/benign ov mass: Sensitivity 84.6% (33/39), specificity 91.1% (112/123), PPV 75% (33/44), *NPV 94.9% (112/118), accuracy 89.5% (145/162) -Advanced OC from controls/benign ov mass: Sensitivity 93.8% (30/32), specificity 91.1% (112/123), PPV 73.2% (30/41), *NPV 98.2% (112/114), accuracy 91.6% (142/155)
Giannopoulou 2017 [31]	Tissue, plasma	Serous (100)	Group A, B: I (19,2), II (57,3), III (18,64), IV (-,13) unk (6,23)	Tissue: Group A: 67 OC Group B: 61 OC Group B: 58 matched adjacent cell-free tissue Plasma: Group B: Subset of 59 OC	Tissue: 16 normal fallopian tube Plasma: 51 healthy controls	Bisulfite modified via EZ-DNA Methylation Gold kit 200; real-time MSP and methylation-sensitive high-resolution melting analysis (ms-HRMA)	*RASSF1A*	Tissue: -41% (52/128) OC methylated via MSP, 43% (55/128) OC methylated via ms-HRMA -29.3% (17/58) adjacent cf tissue methylated via MSP, 36% (21/58) adjacent methylated via ms-HRMA -0% (0/16) normal fallopian tube methylated via MSP and ms-HRMA Plasma: -25.4% (15/59) OC methylated via MSP -0% (0/51) healthy control methylated In matched OC, adjacent tissue, and serum (*n* = 53): -84.9% (45/53) matched OC and tissue methylation with strong agreement -62.3% (33/53) matched OC and serum methylation with slight agreement Correlation between methylation with tumor grade (*p* = 0.04), lymph node metastasis (*p* = 0.04), overall survival (*p* =0.02 with ms-HRMA method on tissue). No correlation between methylation and overall survival for plasma samples. ----------------------- Extrapolated summary for plasma test in differentiating OC from healthy controls: -Sensitivity 25.4% (15/59), specificity 100% (51/51) -PPV 100% (15/15), NPV 53.7% (51/95), accuracy 60% (66/110)
Swellam 2017 [45]	Serum	Serous (60), endometrioid (21), mucinous (19)	I-II (47), III-IV (53)	90 OC	50 benign ov mass 30 healthy controls	Bisulfite modified via EpiTect Fast Bisulfite kit; MSP	*DAPK, OPCML, DLEC1*	*DAPK* methylated in 96.7% (87/90) OC, 40% (20/50) benign, 0% (0/30) controls -Differentiating benign and controls from OC: AUC 0.858 *OPCML* methylated in 97.8% (88/90) OC, 48% (24/50) benign, 0% (0/30) controls -Differentiating benign and controls from OC: AUC 0.839 *DLEC1* methylated in 95.6% (86/90), 40% (20/50) benign, 0% (0/30) controls -Differentiating benign and controls from OC: AUC 0.841 Correlation between methylation with: higher stage for *DAPK, OPCML* (*p* = 0.006, *p* < 0.0001 respectively), higher grade (*p* < 0.0001, *p* < 0.001), and serous pathology (*p* = 0.034, *p* = 0.001); higher stage (*p* = 0.03) and grade (*p* < 0.0001) for *DLEC1* -For early stage, sensitivity 95.2-97.6%, specificity 70–75% for all targets -Methylation markers outperformed CA125 and CEA in sensitivity and specificity** ----------------------- Summary for plasma test in differentiating OC from healthy controls/benign ov mass for: *-DAPK:* Sensitivity 96.7% (87/90), specificity 75% (60/80), * PPV 81.3% (87/107), * NPV 95.2% (60/63), accuracy 86.5% (147/170), AUC 0.858 -*OPCML*: Sensitivity 97.8% (88/90), specificity 70% (56/80), * PPV 78.4% (87/111), * NPV 90.3% (56/62), accuracy 84.7% (144/170), AUC 0.839 -*DLEC1:* Sensitivity 95.6% (86/90), specificity 75% (60/80), *PPV 81.1% (86/106), *NPV 93.8% (60/64), accuracy 85.8% (146/170), AUC 0.841
Wang 2017 [30]	Tissue, plasma	Data unavailable^a^ but extrapolated as: Serous (58), endometrioid (11), mucinous (10), clear cell (10), other (11)	I-II (55), III-IV (45)	71 OC	43 benign ov mass 80 healthy controls	Bisulfite modified via EpiTect kit; nested MSP	Initial investigation with *RUNX3, TFPI2, OPCML;* final analysis with *OPCML*	Plasma: -Diagnostic performance of methylation status to detect overall, early, advanced OC -Sensitivity 90%, 87%, 94%, -Specificity 92%, 92%, 92% -Accuracy 91%, 91%, 92% -All values superior to CA125 -Methylation difference between healthy controls and early, advanced, and overall OC (*p* < 0.0001) -No correlations between methylation with healthy controls and benign tumors -Methylation markers outperformed CA125 in detection of early-stage OC ----------------------- Summary for plasma test in differentiating: -All OC from controls/benign ov mass: Sensitivity 90.1% (64/71), specificity 91.9% (113/123), PPV 86% (64/74), *NPV 94.2% (113/120), accuracy 91.2% (177/194) -Early OC from controls/benign ov mass: Sensitivity 87.2% (34/39), specificity 91.9% (113/123), PPV 77.3% (34/44), ^NPV 95.8% (113/118), accuracy 90.7% (147/162) -Advanced OC from controls/benign ov mass: Sensitivity 93.8% (30/32), specificity 91.9 (113/123), PPV 75% (30/40), *NPV 98.3% (113/115), accuracy 92.3% (143/155)
Widschwendter 2017 [35]	Tissue, serum	Marker discovery Array: Data unavailable RRBS: Serous (82), endometrioid (9), mucinous (9) Assay development: Serous (75), endometrioid (11), mucinous (4), clear cell (9) Assay validation: Data incomplete	Marker discovery: Data unavailable Assay development: I-II (44), III-IV (56) Assay validation: Data incomplete	Marker discovery with 2 tissue sets: Array: 218 OC, 10 benign pelvic mass, 55 fallopian tube, 96 endometrium, 107 WBC, 170 other organs RRBS: 11 OC, 1 benign pelvic mass, 18 normal, 2 endometrium, 23 WBC Assay development with 2 serum sets: 45 OC, 11 borderline, 56 benign pelvic mass, 39 healthy controls Assay validation with 3 serum sets: 164 OC (including 5 non-epithelial), 27 borderline, 119 benign pelvic mass, 37 other cancer, 150 healthy controls		Bisulfite modified at GATC Biotech; RRBS performed at GATC Biotech and GWAS methylation analysis via Infinium Human Methylation 450 K array; PCR with ultra-high coverage bisulfite sequencing via Illumina MiSeq or HiSeq 2500	Four candidate markers for differentiating high-grade serous patients, specifically; narrowed to 3 due to limited serum volume: *COL23A1, C2CD4D, WNT6*	Diagnostic performance of methylation assay in: -Differentiating HGS OC from benign/controls in validation set: Sensitivity 41.4% (12/29), specificity 90.7% (127/140) -Early detection in screened healthy participants (after reducing threshold for regions and evaluating only samples with less than the median amount of DNA): Sensitivity 58%, specificity 88% -Early detection only for CA125-normal healthy participants: Sensitivity 64%, specificity 87.5% -Identifying chemotherapy responders and non-responders, respectively: Sensitivity 78%, 86% Key limitation for screening sample: Leukocyte DNA leakage into serum samples due to delayed processing time ----------------------- Summary for serum test in differentiating HGS OC from: -Benign/controls in validation sample: Sensitivity 41.4% (12/29), specificity 90.7% (127/140), * PPV 48% (12/25), * NPV 88.2% (127/144), * accuracy 82.2% (139/169) -Healthy controls in screening sample: Sensitivity 58% * (25/43), specificity 88% * (114/129), * PPV 62.5% (25/40), * NPV 91.2% (114/125), * accuracy 80.8% (139/172) -Healthy controls in screening sample with normal CA125: Sensitivity 64%, specificity 87.5%, no further data provided for extrapolation
Giannopoulou 2018 [32]	Tissue, plasma	Serous (100)	Group A, B: I (20, 6); II (56, 5); III (21, 76); IV (-,13)	Tissue: Group A: 66 OC Group B: 63 OC Plasma: Group B: Subset of 50 OC (chemo-treated)	Tissue: 16 normal fallopian tube Plasma: 51 healthy controls	Bisulfite modified via EZ-DNA Methylation Gold kit 200; real-time MSP	*ESR1*	Tissue: -39% (47/119) OC methylated -94% (15/16) normal fallopian tube methylated Plasma: -38% (19/50) OC methylated -2% (1/51) healthy control methylated In matched OC and serum: -75% (36/48) matched OC and serum methylation with moderate agreement - No correlations between serum methylation with clinicopathologic features, survival ----------------------- Extrapolated summary for plasma test in differentiating OC from healthy controls: -Sensitivity 38% (19/50), specificity 84.7% (50/51) -PPV 95% (19/20), NPV 61.7% (50/81), accuracy 68.3% (69/101)

Percentages may not add up to 100 given rounding. OC: ovarian cancer; MSP: methylation-specific PCR: ov: ovary/ovarian; unk: unknown; GWAS: genome-wide association study; RRBS: reduced representation bisulfite sequencing; RRSO: risk-reducing salpingo-oophorectomy; HGS: high-grade serous; NS: non-significant. ^a^ Histological data unavailable from this publication but could be determined from the author’s previous publication. * Extrapolated statistical calculations based on available data.

**Table 2:** Gene targets for methylation analysis on cell-free DNA from patient serum or plasma.

Gene Target		Type	Description	Ref.
*RASSF1A*	Ras association domain-containing protein 1	Tumor suppressor	Modulates multiple apoptotic and cell cycle checkpoint functions	[27,28,31,33,34,36,37,44]
*OPCML*	Opioid binding protein/cell adhesion molecule-like gene	Tumor suppressor	Involved in cell adhesion and cell–cell recognition	[29,30,34,43,45]
*BRCA1*	Breast cancer type 1 susceptibility protein	Tumor suppressor	Maintains genomic stability via DNA repair, cell cycle regulation, and others	[28,37,42]
*DAPK*	Death-associated protein kinase 1	Tumor suppressor	Involved in multiple cell death-associated signaling pathways	[37,38,45]
*APC*	Adenomatous polyposis coli	Tumor suppressor	Controls cell division, motility, and adhesion	[34,37]
*RUNX3*	Runt-related transcription factor 3	Tumor suppressor	Involved in cell differentiation and DNA repair	[29,34]
*TFP12*	Tissue factor pathway inhibitor 2	Tumor suppressor	Involved in regulation of extracellular matrix digestion and remodeling	[29,34]
*SFRP5, 1, 2*	Secreted frizzled-related protein 5,1,2	Tumor suppressor	Regulates cell growth and differentiation	[34,40]
*PAX1, 5*	Paired box 1, 5	Oncogene; tumor suppressor	Involved in cell development	[28,40]
*PGR, PGR-PROX*	Progesterone receptor; Progesterone receptor, proximal promotor	Oncogene; tumor suppressor	Regulates cell proliferation and differentiation	[27,28]
*p14-ARF*	Alternate reading frame protein product	Tumor suppressor	Involved in cell cycle regulation and apoptosis	[37]
*p16-INK4a*	Inhibitors of CDK4	Tumor suppressor	Regulates cell cycle progression	[37]
*H1C1*	Hypermethylated in cancer 1	Tumor suppressor	Regulates cell growth and apoptosis	[28]
*THBS1*	Thrombospondin 1	Tumor suppressor	Modulates cell motility, adhesion, and growth	[28]
*EP300*	Adenovirus early region 1A-associated protein p300	Tumor suppressor	Regulates cell growth and differentiation	[27]
*CALCA*	Calcitonin related polypeptide alpha	Tumor suppressor	Regulates calcium and phosphorous metabolism; vasodilation	[27]
*SLIT2*	Slit guidance ligand 2	Tumor suppressor	Involved in cell migration processes	[41]
*SOX1*	Sex determining region Y-box 1	Tumor suppressor	Regulates embryonic development and stem cell function	[40]
*LMX1A*	LIM homeobox transcription factor 1, alpha	Tumor suppressor	Regulates cell growth and differentiation	[40]
*CDH1*	Cadherin-1	Tumor suppressor	Involved with cell adhesion and motility	[34]
*PTEN*	Phosphatase and tensin homolog	Tumor suppressor	Regulates cell proliferation	[44]
*ESR1*	Estrogen receptor, alpha 1	Oncogene; tumor suppressor	Regulates cell proliferation and differentiation	[32]
*WNT6*	Wingless-type MMTV integration site family, member 6	Oncogene	Involved with cell proliferation, differentiation, adhesion	[35]
*COL23A1*	Collagen, type XIII, alpha 1	Oncogene	Likely involved with cell adhesion	[35]
*C2CD4D*	C2 calcium-dependent domain containing 4D	Oncogene	Unknown	[35]
*DLEC1*	Deleted in lung and esophageal cancer1	Tumor suppressor	Regulates cell proliferation	[45]

**Table 3:** Review search terms. Two searches were conducted in PubMed’s Medline and Elsevier’s Embase and Scopus. MeSH terms were used only in Medline. Terms were combined using Boolean logic statements: Within-column terms were combined with “OR”; between-column terms were combined using “AND”. The asterisk (*) represents a wildcard symbol used to broaden the search to all terms with the previous stem.

Search Number	Early Diagnosis	Ovarian Cancer	Biomarkers	Type of Marker
**1**	Early diagnosis [MeSH]	Ovarian neoplasms [MeSH]	Biomarkers, Tumor [MeSH]	DNA methylation
	Early diagnosis	Ovarian neoplasms/diagnosis [MeSH]	Biomarker*	ctDNA*
	Early detection of cancer [MeSH]	Ovarian neoplasms	Tumor marker*	Circulating tumor DNA
	Cancer screening	Ovarian cancer	Tumor biomarkers	miRNA*
		High-grade serous carcinoma	Cancer biomarkers	microRNA
		HGSC	Neoplasm markers	Cytolog*
		Cystadenocarcinoma, Serous [MeSH]	Carcinogen markers	
		Serous cystadenoma		
		Serous epithelial		
**2**	Early diagnosis [MeSH]	Ovarian neoplasms [MeSH]		ctDNA*
	Early diagnosis	Ovarian neoplasms/diagnosis [MeSH]		Circulating tumor DNA
	Early detection of cancer [MeSH]	Ovarian neoplasms		cfDNA*
	Cancer screening	Ovarian cancer		Cell free DNA
		High-grade serous carcinoma		Liquid biopsy
		HGSC		Liquid biopsies
		Cystadenocarcinoma, Serous [MeSH]		Methylation
		Serous cystadenoma		Epigenetic
		Serous epithelial		

## Data Availability

No new data were created or analyzed in this study. Data sharing is not applicable to this article.

## Supplementary Materials

The following are available online at https://www.mdpi.com/2072-6694/13/4/838/s1, Table S1: Sensitivities and specificities for each target gene in discriminating between ovarian cancer with non-ovarian cancer specimens. Genes included in panels are represented by the test characteristics of the panel. If a gene was included in more than one study, the median and range is reported. Otherwise, values are percentages, Table S2: Preferred Reporting Items for Systematic reviews and Meta-Analyses extension for Scoping Reviews (PRISMA-ScR) Checklist.
